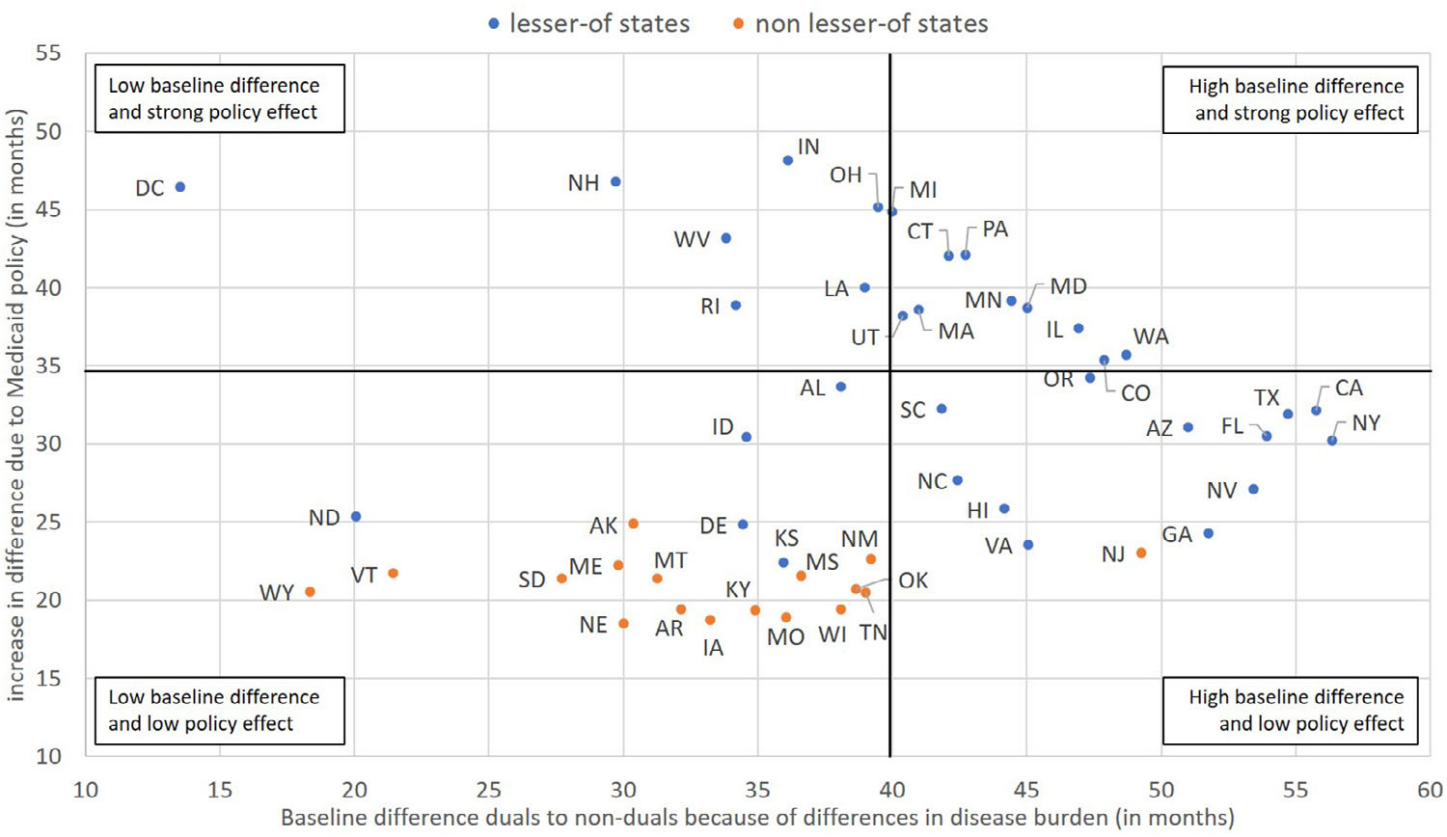# Projected differences in wait times for diagnosis of Alzheimer’s disease for individuals dually eligible for Medicare and Medicaid in the U.S due to higher disease burden and obstacles to access

**DOI:** 10.1002/alz.084598

**Published:** 2025-01-09

**Authors:** Soeren Mattke, Samantha Chu, Hankyung Jun, Mark Hanson

**Affiliations:** ^1^ University of Southern California, Los Angeles, CA USA; ^2^ Cornell University, Ithaca, NY USA; ^3^ Harvard Medical School, Boston, MA USA

## Abstract

**Background:**

As disease‐modifying treatments for Alzheimer’s disease (AD) are becoming available in the US, concerns have been raised that an unprepared healthcare system will be unable to cope with the expected influx of patients. Individuals dually eligible for Medicare and Medicaid might be disproportionately affected by wait times for three reasons. They have higher burden of disease; many practices do not accept Medicaid at all and those who do might limit the number of Medicaid patients because of lower payment rates under the so‐called lesser‐of policy. States with that policy may elect to pay only up to the Medicaid rate when covering Medicare cost‐sharing requirements for their indigent members. We estimate the effect of the three types of obstacles on wait times.

**Method:**

We used a Markov model to predict wait times for dually eligible and regular Medicare beneficiaries in all 50 states and the District of Columbia from 2023 to 2050. Counts of both groups by age, sex and states were derived from Census data and Medicare enrollment records. We obtained Medicaid rates, estimates for price elasticity of supply of specialty services, willingness of practices to accept Medicaid and burden of disease from published data.

**Result:**

Higher disease burden in duals is projected to lead to wait times of 59.8 months compared to 20.7 months for non‐duals even without considering Medicaid‐specific obstacles to access. Assuming 25% of specialists would not accept Medicaid at all would add around 21.9 months on average. As Medicaid payment rates differ by state, their effect on wait times is projected to vary substantially from adding 2.2 months on average in Virginia to adding at least 99 months in 14 states, such as West Virginia, Oregon and Pennsylvania. Figure 1 illustrates the interaction of disease burden and policy effect by state.

**Conclusion:**

The socio‐economically disadvantaged group of duals might face disproportionate wait times for an AD diagnosis, which could be long enough to deprive them of access to treatment entirely and translate into avoidable disease progression and mortality, raising concerns about health equity.